# Ginkgolide B promotes neuronal differentiation through the Wnt/β-catenin pathway in neural stem cells of the postnatal mammalian subventricular zone

**DOI:** 10.1038/s41598-018-32960-8

**Published:** 2018-10-08

**Authors:** Ming-Yang Li, Chia-Ting Chang, Yueh-Ting Han, Chien-Po Liao, Jenn-Yah Yu, Tsu-Wei Wang

**Affiliations:** 1 Department of Life Science, National Taiwan Normal University, Taipei, 116 Taiwan; 2 Department of Life Sciences and Institute of Genome Sciences, National Yang-Ming University, Taipei, 112 Taiwan; 3 Brain Research Center, National Yang-Ming University, Taipei, 112 Taiwan

## Abstract

Chinese herbal medicines (CHMs) have been used to treat human diseases for thousands of years. Among them, *Ginkgo biloba* is reported to be beneficial to the nervous system and a potential treatment of neurological disorders. Since the presence of adult neural stem cells (NSCs) brings hope that the brain may heal itself, whether the effect of *Ginkgo biloba* is on NSCs remains elusive. In this study, we found that *Ginkgo biloba* extract (GBE) and one of its main ingredients, ginkgolide B (GB) promoted cell cycle exit and neuronal differentiation in NSCs derived from the postnatal subventricular zone (SVZ) of the mouse lateral ventricle. Furthermore, the administration of GB increased the nuclear level of β-catenin and activated the canonical Wnt pathway. Knockdown of β-catenin blocked the neurogenic effect of GB, suggesting that GB promotes neuronal differentiation through the Wnt/β-catenin pathway. Thus, our data provide a potential mechanism underlying the therapeutic effect of GBE or GB on brain injuries and neurodegenerative disorders.

## Introduction

In mammals, neural stem cells (NSCs) in the subventricular zone (SVZ) of the lateral ventricle and the subgranule zone (SGZ) of the hippocampal dentate gyrus (DG) give rise to new neurons in the olfactory bulb (OB) and DG throughout adulthood, respectively^1^. In addition, adult striatal neurogenesis has been discovered in humans^2^. Importantly, postnatal neurogenesis is induced or increased in the injured cerebral cortex, hippocampus or striatum^3–7^, which are also vulnerable in various neurodegenerative disorders such as Alzheimer’s disease (AD) and Huntington’s disease (HD). Therefore, strategies to enhance neurogenesis of endogenous NSCs could be a promising therapeutic treatment for relieving brain injuries or neurodegenerative disorders.

In the SVZ, NSCs undergo self-renew and generate transit-amplifying cells, which give rise to neuroblasts. Neuroblasts migrate along the rostral migratory stream (RMS) to the OB and then differentiate into mature neurons^1^. Many signaling pathways, such as Notch, Sonic Hedgehog (Shh), Wnt/β-catenin and extracellular signal-regulated kinase (ERK) pathways activated by neurotrophic factors have been demonstrated to regulate self-renewal and neurogenesis of NSCs^8–12^. Interestingly, components of Chinese herbal medicines (CHMs), such as baicalin or curcumin, are shown to induce neurogenesis through these pathways^13,14^. Since CHMs have been shown to be beneficial to various neurological diseases, such as AD and HD, it prompts us to screen CHMs and components of CHMs for promoting neurogenesis.

Among CHMs, *Ginkgo biloba* extract (GBE) has been demonstrated to alleviate symptoms of age-related dementia, AD and ischemia^15–17^. It has also been shown that GBE improves spatial learning and/or memory in young rats and a transgenic mouse model of AD^18,19^. Several cellular and molecular mechanisms underlying therapeutic effects of GBE are emerging. GBE may function as a free-radical scavenger to attenuate oxidative stress^20^. It has also been suggested that GBE prevents cell death and promotes hippocampal neurogenesis through stimulating phosphorylation of cyclic-AMP response element binding protein (CREB) and elevation of brain-derived neurotrophic factor (BDNF)^21–25^. A standardized extract of GBE contains approximately 24% of flavonoid glycosides (primarily quercetin, kaempferol and isorhamnetin) and 6% of terpenoids (2.8–3.4% of which are ginkgolide (G) A, B and C, a few of GJ and 2.6–3.2% of bilobalide)^20^. Therefore, it is also important to identify the effective components in GBE for treating neurological disorders.

Although GBE has been demonstrated to have positive effects on the nervous system, whether it also affects NSCs and the underlying mechanism have not been thoroughly studied. Here, we investigated the neurogenic effect of GBE. We found that both GBE and GB promoted neuronal differentiation in postnatal NSCs. Importantly, the neurogenic effect of GB was mediated by the canonical Wnt/β-catenin pathway. Together, our data reveal a mechanism of GBE and GB in regulating postnatal neurogenesis in mammalian brains.

## Results

### GBE promotes neuronal differentiation in P19 cells

We first used P19 cells as a model to test the effect of GBE on neuronal differentiation. P19 mouse carcinoma cell line can be induced to differentiate into neural cells or myocytes under appropriate conditions, which serves a good model to screen for potential neurogenic compounds^26,27^. Retinoic acid (RA) treatment of P19 cell aggregates results in neuronal differentiation^27^. We first investigated whether GBE promoted neuronal differentiation of P19 cells after RA-induced neuronal induction. P19 cells were cultured as aggregates with RA for four days and then cultured in monolayer with GBE (1 mg/ml) for another three days. Neuronal differentiation was examined by immunofluorescence with Tuj1, an antibody recognizing neuronal βIII-tubulin^26^. GBE significantly increased the number of Tuj1-positive cells (Ctrl: 100 ± 5%, GBE: 123.5 ± 6.1%, p < 0.05; Fig. S1A–C). This result indicates that GBE facilitates neuronal differentiation in P19 cells.

To further confirm the neurogenic effect of GBE, P19 cells were grown in adherent culture with different concentrations (1 ng/ml, 1 µg/ml or 1 mg/ml) of GBE without RA treatment for three days. 1 µg/ml or 1 mg/ml, but not 1 ng/ml of GBE significantly increased the number of Tuj1-positive cells (Ctrl: 6.9 ± 1.2 cells/mm^2^, 1 ng/ml: 9.5 ± 1.5 cells/mm^2^, n.s.; 1 µg/ml: 12.1 ± 1.5 cells/mm^2^, p < 0.05; 1 mg/ml: 13 ± 1.2 cells/mm^2^, p < 0.05; Fig. S1D). This result demonstrates a dose-dependent effect of GBE on promoting neuronal differentiation. An alternative explanation is that GBE may promote cell survival, which in turn increases the number of Tuj1-positive neurons. Since total cell numbers in all groups were comparable (Ctrl: 1088.5 ± 121.1 cells/mm^2^, 1 ng/ml: 1078.6 ± 113.8 cells/mm^2^, 1 µg/ml: 1040.3 ± 114 cells/mm^2^, 1 mg/ml: 1112.3 ± 42.6 cells/mm^2^, n.s.), it is less likely that GBE increases the number of neurons through promoting cell viability in our cultural condition.

### GBE promotes neuronal differentiation in postnatal NSCs

**Figure 1:**
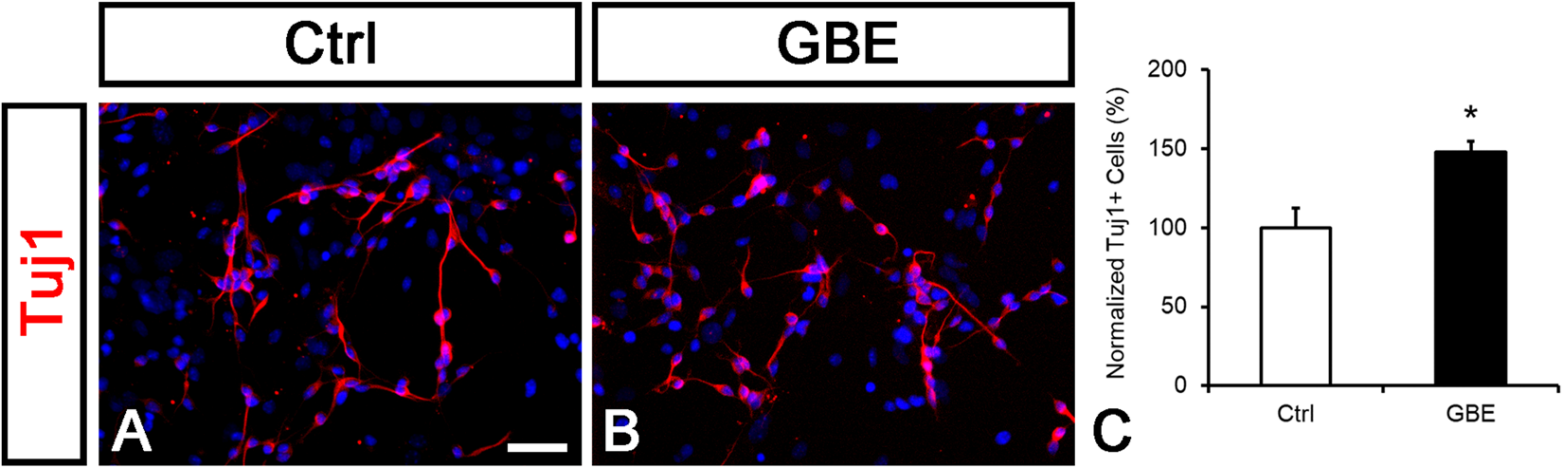
GBE promotes neuronal differentiation in postnatal NSCs. (**A**,**B**) NSCs derived from P7 mice were cultured to form 1′ NSs and then grown in the differentiation condition with vehicle (Ctrl) or 1 mg/ml of GBE for three days. Cells were immunolabeled with Tuj1 antibodies in red. Nuclear DNA was stained with DAPI in blue. (**C**) Tuj1-positive cell numbers were counted. GBE treatment significantly increased the number of Tuj1-positive cells. The differentiation rate was normalized to the control. n = 3, *p < 0.05 compared to the control; t-test. Data are shown as mean ± SEM. Scale bar: 50 µm.

Since P19 cell is an immortalized cell line, we further examined whether GBE induced neuronal differentiation in NSCs isolated from the postnatal SVZ. Neurosphere culture is a well-established method to study NSC properties *in vitro*^28^. Cells from the SVZ of the lateral ventricle in postnatal day 7 (P7) mice were cultured in suspension condition with EGF and FGF to form primary neurospheres (1′NSs). After cultured *in vitro* for five days, 1′NSs were dissociated and plated with differentiation medium supplemented with GBE (1 mg/ml). The administration of GBE significantly increased Tuj1-positive cells compared with those in the control group (Ctrl: 100 ± 12.4%, GBE: 148.0 ± 6.5%, p < 0.05; Fig. 1). This result suggests that GBE promotes neuronal differentiation in NSCs derived from the postnatal SVZ.

### GB promotes neuronal differentiation in postnatal NSCs

**Figure 2:**
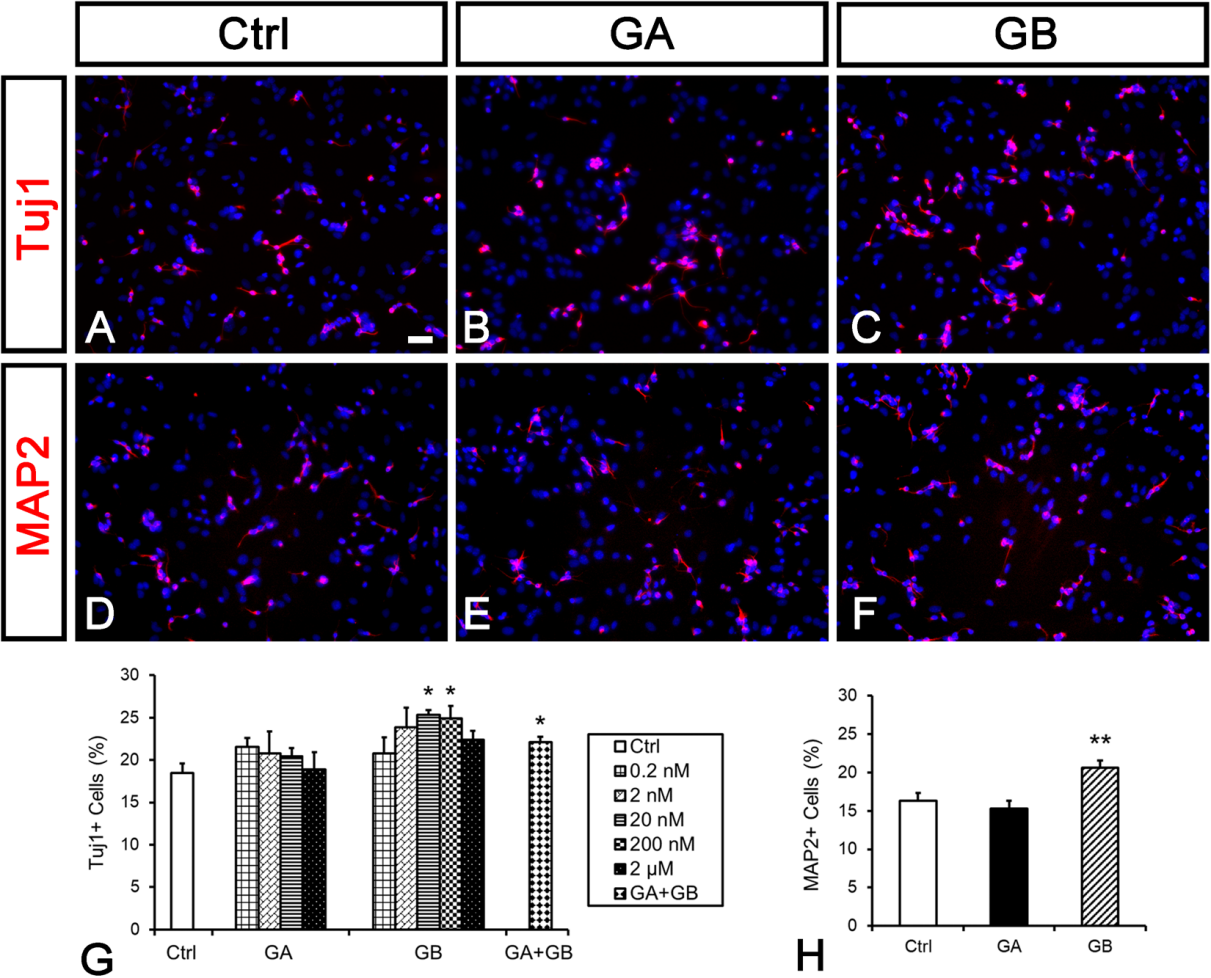
GB promotes neuronal differentiation in postnatal NSCs. NSCs derived from P7 mice were cultured to form 1′ NSs and then grown in the differentiation condition with vehicle (Ctrl) or 0.2 nM to 2 µM of GA or GB for three days. Cells were immunolabeled with Tuj1 or MAP2 antibodies in red. Nuclear DNA was stained with DAPI in blue. (**A**–**F**) Representative images of Tuj1 (**A**–**C**) and MAP2 (**D**–**F**) labeling from Ctrl (**A**,**D**), 20 nM of GA (**B**,**E**), and 20 nM of GB (**C**,**F**) groups. (**G**) Quantification of Tuj1-positive cells. GA did not increase the percentage of Tuj1-positive cells over total cells. 20 and 200 nM of GB increased the percentage of Tuj1-positive cells over total cells. 20 nM GA plus GB increased Tuj1-positive cells over total cells. (**H**) Quantification of MAP2-positive cells. 20 nM of GB, but not GA, increased the percentage of MAP2-positive cells over total cells. n ≥ 3. Data are shown as mean ± SEM. *p < 0.05, **p < 0.01 compared to the control; one-way ANOVA followed by Tukey’s *post hoc* test. Scale bar: 50 µm.

GBE contains 5–7% of terpenoids and 2.8–3.4% of which are GA, B, C and J^20^. GA, GB, and GJ have been reported to protect neurons or preserve synaptic functions in AD models^29,30^. Since we found that 1 µg-1mg/ml of GBE promoted neuronal differentiation in P19 cells and postnatal NSCs, the comparable concentration of GA (MW = 408.4) and GB (MW = 424.4) should be between 4.4 nM–4.4 µM and 5.6 nM–5.6 µM, respectively. We tested whether the administration of or GA or GB could induce neuronal differentiation in postnatal NSCs at various concentrations (0.2 nM to 2 µM). No significant difference was observed in the percentage of Tuj1-positive cells with GA treatments (Ctrl: 18.5 ± 1.1%, 0.2 nM: 21.6 ± 1%, 2 nM: 20.8 ± 2.3%, 20 nM: 20.4 ± 1%, 2 µM: 18.9 ± 2.1%, n.s.; Fig. 2A,B,G). Importantly, 20 nM and 200 nM of GB promoted neuronal differentiation in NSCs (Ctrl: 18.5 ± 1.1%, 0.2 nM: 20.8 ± 1.9%, n.s.; 2 nM: 23.9 ± 2.3%, n.s.; 20 nM: 25.3 ± 0.6%, p < 0.05; 200 nM: 24.9 ± 1.4%, p < 0.05; 2 µM: 22.4 ± 1%, n.s.; Fig. 2A,C,G). We used another neuronal marker MAP2 to confirm the neurogenic effect of GB by performing immunofluorescence three days after 20 nM of GA or GB treatments in postnatal NSCs. The percentage of MAP2-positive cells in GA group was similar to that in the control, but was increased in GB group (Ctrl: 16.3 ± 1%, GA: 15.3 ± 0.2%, n.s.; GB: 20.6 ± 0.9%, p < 0.01; Fig. 2D,E,F,H). These results suggest that GB may be a key component in GBE to promote neuronal differentiation.

**Figure 3:**
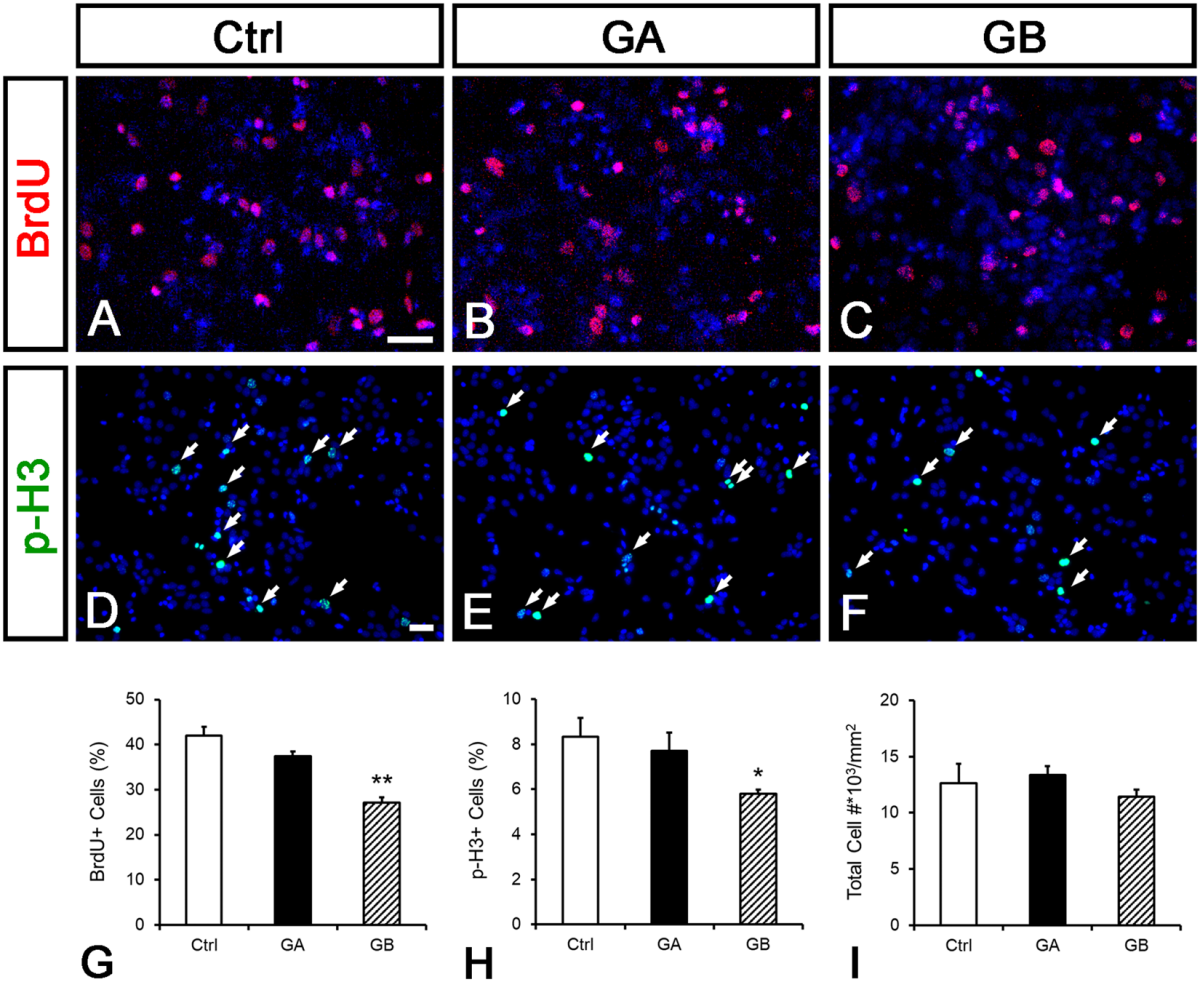
GB inhibits cell proliferation in postnatal NSCs. Postnatal NSCs were cultured in the differentiation condition with vehicle, 20 nM of GA or GB for two days. BrdU was added two hours before fixation. Cells were immunolabeled with BrdU antibodies in red or p-H3 in green. Nuclear DNA was stained with DAPI in blue. (**A**–**F**) Representative images of BrdU (**A**–**C**) and p-H3 (**D**–**F**) labeling from Ctrl (**A**,**D**), 20 nM of GA (**B**,**E**), and 20 nM of GB (**C**,**F**) groups. (**G**,**H**) Quantification of BrdU- and p-H3-positive cell numbers. GB decreased the percentage of BrdU- and p-H3-positive cells over total cells. (**I**) There was no difference in total cell number in these three groups. n = 3. Data are shown as mean ± SEM. *p < 0.05, **p < 0.01 compared to the control; one-way ANOVA followed by Tukey’s *post hoc* test. Scale bar: 50 µm.

We further examined whether GB affected cell proliferation by using BrdU incorporation. Two hours before fixation, BrdU was added into postnatal NSC cultures that had been treated with 20 nM of GA or GB for two days. Cells incorporated BrdU were detected by immunofluorescence with BrdU antibody. The percentage of BrdU-positive cells was significantly decreased with GB but not GA treatment in comparison with that of the control group (Ctrl: 42 ± 2%, GA: 37.4 ± 1.1%, n.s.; GB: 27.2 ± 1.2%, p < 0.01; Fig. 3A–C,G). We also used the mitotic marker pH3 to confirm that GB but not GA induced cell cycle exit. Postnatal NSCs were cultured with 20 nM GA or GB for two days and labeled with antibody against pH3. Consistently, the percentage of pH3-positive cells in GA group was similar to that in the control, but was decreased with GB treatment (Ctrl: 8.3 ± 0.8%, GA: 7.7 ± 0.8%, n.s.; GB: 5.8 ± 0.2%, p < 0.05; Fig. 3D–F,H). These data suggest that GB inhibits cell proliferation. Since neuronal differentiation starts after cell cycle withdraw^31,32^, our results demonstrate that GB may promote cell cycle exit to induce neuronal differentiation.

An alternative explanation is that GB promotes cell survival, which in turn increases the number Tuj1 and MAP2-positive neurons. To examine whether GB regulates apoptosis, postnatal NSCs were cultured in the differentiation condition with 20 nM GA or GB for three days and immunolabeled with cleaved Caspase-3 antibody. The percentage of cleaved Caspase-3-positive cells in both GA and GB groups were similar to that in control (Ctrl: 0.3 ± 0.2%, GA: 0.5 ± 0.1%, GB: 0.3 ± 0.1%, n.s.). Consistently, total cell number was not changed in GA or GB treatment (Ctrl: 12631.4 ± 2891.1/mm^2^, GA: 13383 ± 785.2/mm^2^, GB: 11409.3 ± 627.2/mm^2^, n.s.; Fig. 3I). Taken together, these results suggest that GB promotes neuronal differentiation instead of affecting cell viability.

### GB increases the level of nuclear β-catenin and activates the Wnt pathway

**Figure 4:**
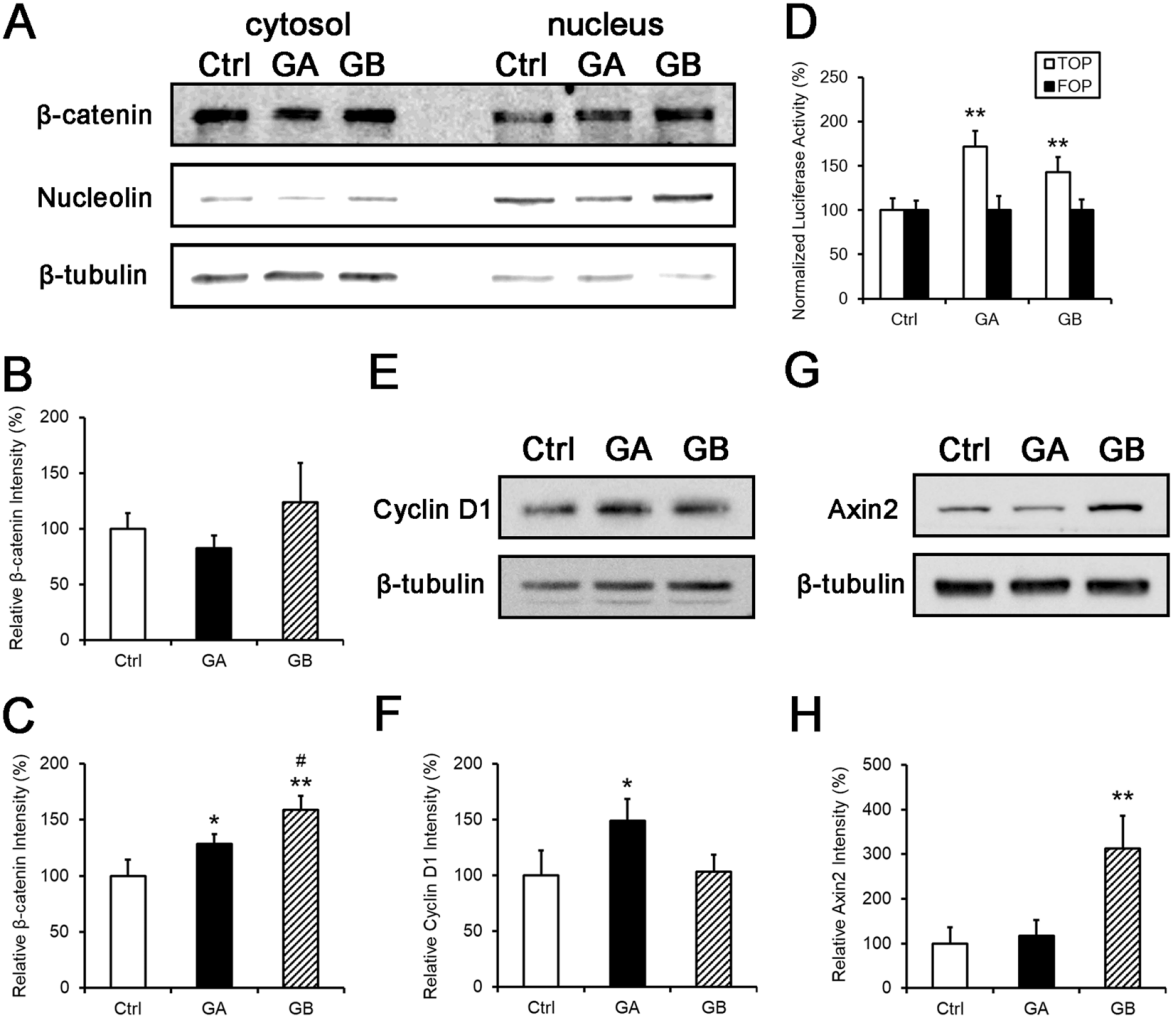
GB increases nuclear β-catenin and activate the Wnt pathway. (**A**–**C**) P19 cells were lysed three hours after GA or GB treatment and cytosolic and nuclear proteins were subjected to Western blot analysis for β-catenin. β-tubulin (for cytosol)/Nucleolin (for nucleus) was used as a loading control. (**B**) Quantification of cytosolic β-catenin. The intensity of cytosolic β-catenin was normalized to that of β-tubulin and results were shown as relative intensity to the control. There was no difference in the cytosolic β-catenin level between groups. (**C**) Quantification of nuclear β-catenin. The intensity of nuclear β-catenin was normalized to that of Nucleolin and results were shown as relative intensity to the control. GA and GB significantly increased nuclear β-catenin and GB had a stronger effect than GA. n = 4. (**D**) P19 cells were transfected with *firefly Luciferase* reporter constructs with either wild-type (TOP) or mutant (FOP) TCF/LEF binding sites. The *US2-renilla Luciferase* construct was co-transfected in each group and its activity was served as an internal control. Luciferase activities were measured 24 hours after transfection. Normalized Luciferase activities are shown as mean ± SEM. GA and GB significantly increased the Luciferase activity in cells transfected with TOP sites but not with FOP sites. n = 6. (**E**–**H**) P19 cells were lysed 24 hours after GA or GB treatment and proteins were subjected to Western blot analysis for Cyclin D1 (**E**) and Axin2 (**G**). β-tubulin was used as a loading control. (**F**) Quantification of Cyclin D1. The intensity of Cyclin D1 was normalized to that of β-tubulin and results were shown as relative intensity to the control. GA significantly increased Cyclin D1 expression. (**H**) Quantification of Axin2. The intensity of Axin2 was normalized to that of β-tubulin and results were shown as relative intensity to the control. GB significantly increased Axin2 expression. n ≥ 3. *; ^#^*p* < 0.05, **p < 0.01. *Compared to the control; ^#^compared to GA; one-way or two-way ANOVA fallowed by Tukey’s *post hoc* test.

Since we found that GBE increased neuronal differentiation both in P19 cells and postnatal NSCs and P19 cells are considered as a good cell model for biochemical studies, we used them to study the downstream signaling pathways induced by GBE or GB. Both ERK and Wnt pathways are important for regulating neurogenesis, neurite outgrowth and neuronal survival^9–12^. P19 cells were lysed 24 hours after GBE treatment. The ratio of pERK over total ERK was used as an index for ERK activity. GBE treatment did not affect the activity of ERK (Ctrl: 100 ± 30%, GBE: 94.1 ± 9.5%, n.s.), suggesting that GBE may not promote neuronal differentiation through the ERK pathway. Since the activation of the canonical Wnt pathway leads to nuclear translocation of the effector protein β-catenin, we performed nuclear extraction to obtain fractions enriched in cytosol and nuclear proteins. The level of β-catenin was unchanged in the cytosol after 20 nM of GA or GB treatment (Ctrl: 100 ± 14.1%, GA: 82.2 ± 11.9%, n.s.; GB: 123.6 ± 35.6%, n.s.; Fig. 4A,B), but was significantly increased in the nucleus in the GA and GB group, and GB had a stronger effect than GA (Ctrl: 100 ± 14.4%, GA: 128.7 ± 8.5%, p < 0.05; GB: 159.1 ± 12.5%, p < 0.01 compared to Ctrl, p < 0.05 compared to GA; Fig. 4A,C). This result suggests that both GA and GB activate the canonical Wnt pathway, but not the ERK pathway.

We further tested whether increased nuclear β-catenin by GA and GB enhanced the activity of canonical Wnt pathway. Activation of the Wnt pathway promotes the transcription activity of TCF/LEF. P19 cells were transfected with firefly Luciferase reporter constructs with either wild-type (TOP) or mutant (FOP) TCF/LEF binding sites and treated with GA or GB. The *US2-renilla Luciferase* construct was co-transfected for all groups and the activity of renilla Luciferase served as an internal control. Luciferase activities were measured 24 hours after transfection. Both GA and GB increased the Luciferase activity with TOP but not FOP (Ctrl: 100 ± 13.4%, GA: 171.5 ± 27.2%, p < 0.01; GB: 143 ± 16.6%, p < 0.01; Fig. 4D), demonstrating that both GA and GB activate the canonical Wnt pathway.

To confirm that the Wnt pathway was indeed induced by GA and GB, we also examined the expression of the Wnt target genes, Cyclin D1 and Axin2^33^. Cyclin D1 was significantly increased in P19 cells after GA but not GB treatment (Ctrl: 100 ± 22.5%, GA: 148.8 ± 20.0%, p < 0.05; GB: 103.3 ± 15.3%, n.s.; Fig. 4E,F). On the other hand, Axin2 was significantly increased in P19 cells after GB but not GA treatment (Ctrl: 100 ± 36.1%, GA: 116.5 ± 35.2%, n.s.; GB: 313 ± 72.7%, p < 0.01; Fig. 4G,H). A similar result was also found in postnatal NSCs after GA and GB treatment (data not shown). Taken together, our data suggest that both GA and GB activate the Wnt pathway. Interestingly, GA and GB induce the expression of different target genes and hence may result in the divergent outcome in cell proliferation and neuronal differentiation of postnatal NSCs.

We have demonstrated that GBE (contained both GA and GB) induced neuronal differentiation in postnatal NSCs (Fig. 1). However, GB but not GA increased neuronal differentiation (Fig. 2) and GA but not GB maintained cell proliferation (Fig. 3). Since only GA induced the expression of Cyclin D1, a well known regulator of cell cycle and knockout of *Cyclin D1* decreases cell proliferation in the adult SVZ^34^, it is possible that cell cycle exit promoted by GB is the main driving force for neuronal differentiation. When postnatal NSCs are treated with both GA and GB, the effect of GB on cell cycle exit wins out, which in turn drives neuronal differentiation. To examine this hypothesis, postnatal NSCs from dissociated 1′ NSs were simultaneously treated with 20 nM of GA and GB for three days and immunolabeled with Tuj1 antibody. GA plus GB significantly increased neuronal differentiation compared with that in the control group (GA + GB: 22.1 ± 0.6%, p < 0.05; Fig. 2G). Together with that GA and GB induced the expression of different Wnt target genes (Fig. 4F,H), these results suggest that GB is the main neurogenic factor in GBE.

### GB promotes neuronal differentiation through the Wnt pathway in postnatal NSCs

**Figure 5:**
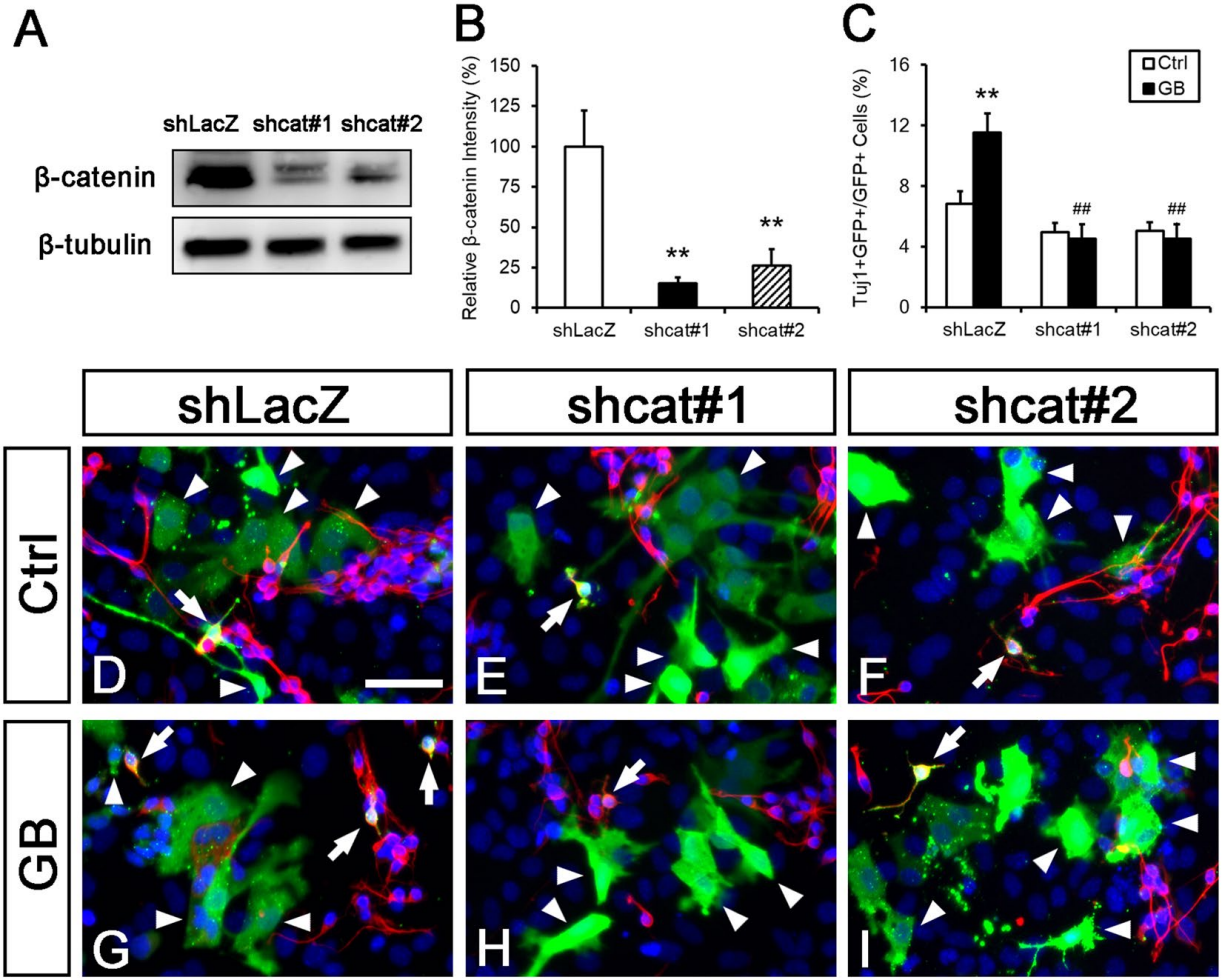
GB promotes neuronal differentiation through the Wnt pathway in postnatal NSCs. (**A**) P19 cells were lysed three days after transfection with *shLacZ*, *shcat#1* or *shcat#2* and total proteins were subjected to Western blot analysis for β-catenin. β-tubulin was served as a control. (**B**) Quantification of β-catenin. The intensity of β-catenin was normalized to β-tubulin and the results were shown as relative to the control. Transfection of *shcat#1* and *shcat#2* significantly decreased the relative level of β-catenin. (**C**–**I**) NSCs derived from 1’ NS were transfected with *shcat#1*, *#2* or *shLacZ* as control. Transfected cells were cultured in the differentiation condition with vehicle (**D**–**F**) or 20 nM of GB (**G**–**I**) for three days. A *gfp* expression construct was co-transfected to label transfected cells (green). Cells were immunolabeled with Tuj1in red. Arrows: GFP- and Tuj1-double positive cells; arrowheads: GFP-positive and Tuj1-negative cells. Nuclei were stained with DAPI in blue. (**C**) Quantification of GFP- and Tuj1-double positive cell numbers in D-I. GB promoted neuronal differentiation in postnatal NSCs. Knockdown of *β-catenin* blocked the neurogenic effect of GB. Ctrl: n ≥ 3. The percentage of GFP- and Tuj1-double-positive cells is shown as mean ± SEM. **p < 0.01 compared to the control group; ^##^*p* < 0.01 compared to GB plus *shLacZ*; two-way ANOVA followed by Tukey’s *post hoc* test. Scale bar: 50 µm.

Since GB treatment activates the Wnt signaling and promotes neuronal differentiation, we tested whether GB promoted neuronal differentiation through activating the Wnt pathway. We confirmed the knockdown efficiency of *shcat#1* and *shcat#2*, two shRNA constructs targeting *β-catenin* in P19 cells (shLacZ: 100 ± 22.4%, shcat#1: 15.1 ± 5.6%, p < 0.01; shcat#2: 26.4 ± 10.2%, p < 0.01; Fig. 5A,B). Postnatal NSCs derived from 1′ NSs were transfected with *shcat#1*, *shcat#2* or *shLacZ* (control) together with a *gfp* expression construct to label transfected cells. Transfected cells were then cultured for three days in the differentiation medium supplemented with 20 nM of GB. Consistent with our previous result (Fig. 2), GB promoted neuronal differentiation in postnatal NSCs (Ctrl + shLacZ: 6.8 ± 0.8%, GB + shLacZ: 11.5 ± 1.3%, p < 0.01; Fig. 5C,D,G). Knockdown of *β-catenin* alone did not attenuate neuronal differentiation significantly (Ctrl + shcat#1: 4.9 ± 0.6%, Ctrl + shcat#2: 5 ± 0.6%, n.s.; Fig. 5C–F). This might be due to low endogenous activity of Wnt pathway in our cultural condition. Importantly, knockdown of *β-catenin* blocked neuronal differentiation promoted by GB specifically in transfected GFP-positive cells, but not in GFP-negative ones (GB + shcat#1: 4.5 ± 1%, p < 0.01; GB + shcat#2: 4.5 ± 1%, p < 0.01; Fig. 5C,H,I). This result suggested that GB promotes neuronal differentiation in postnatal NSCs by activating the Wnt pathway in a cell-autonomous manner.

## Discussion

In this study, we reveal that GBE and GB, a component of GBE, promote neuronal differentiation through the Wnt pathway in postnatal NSCs. Therefore, supplement of GBE and GB might be beneficial for brain injuries and neurodegenerative disorders.

Given that cell cycle exit is a key step prior to neuronal differentiation^31,32^, neurogenic factors usually reduce cell proliferation. Indeed, we showed that GB promoted cell cycle withdrawal and induced neuronal differentiation (Figs 2 and 3). It has been shown that GBE increases cell proliferation in NSCs of the SGZ and bone marrow-derived endothelial progenitor cells accompanied by phosphorylation of CREB or activation of AKT/ERK pathway^22–25,35^. In P19 cells or NSCs derived from the postnatal SVZ, however, the Wnt but not ERK pathway acted downstream of GBE/GB in promoting neuronal differentiation (Figs 4 and 5). The Wnt pathway has been shown to induce expression of *NeuroD1*, which in turn promotes cell cycle exit and neuronal differentiation^12,26^. Although we did not find a significant difference of *NeuroD1* expression in postnatal NSCs after GB treatment for two days (data not shown), it is possible that GB induces *NeuroD1* expression earlier. Since NSs grow slowly, we were not able to collect enough cells for Western blot analysis at earlier time points. The other possibility is that it may be caused by a negative feedback control of the Wnt pathway. Activation of Wnt pathway results in an increase of the negative regulator Axin2^33^, which suppresses the activity of the Wnt pathway. We did find an increase of Axin2 expression after GB treatment in P19 cells (Fig. 4G,H), which may explain why we did not observe a significant induction of *NeuroD1* after GB treatment or Wnt activation. Still, we do not rule out the possibility that other target genes of the Wnt pathway or other yet to be identified signaling pathways might account for the neurogenic effect of GB (see below). Nevertheless, different signaling pathways or cellular context may contribute to the divergent effects of GBE and GB on cell proliferation and differentiation in different cell types.

GA and GB are terpenoids from *Ginkgo biloba*. Here we found that both GA and GB activated the Wnt/β-catenin pathway (Fig. 4A–D). Interestingly, GA induced the expression of Cyclin D1 (Fig. 4E,F) and maintained postnatal NSCs in cell cycle (Fig. 3), whereas GB induced the expression of Axin2 (Fig. 4G,H), induced cell cycle exit (Fig. 3) and neuronal differentiation (Fig. 2). How the activation of Wnt pathway by GA and GB results in the induction of different target genes requires further investigation. It is possible that GA and GB may regulate other pathways in addition to the Wnt signaling. In addition to the Wnt signaling, GB has been reported to inhibit the AKT pathway, whereas GA activates it^36,37^. Since the activation of AKT pathway usually promotes progenitor cell proliferation^22–25,35^, it may contribute to the different effects of GB versus GA on cell proliferation and neuronal differentiation.

We found that GB increased nuclear β-catenin and activated the Wnt pathway (Fig. 4). Stabilization of β-catenin and the activation of the canonical Wnt pathway are resulted from the inhibition of GSK-3β^35^. Bilobalide, another component in GBE, has recently been shown to promote phosphorylation and inhibit GSK-3β^38^. Thus, it is possible that GB increase β-catenin through GSK-3β inhibition. Bilobalide also induces the expression of Wnt ligands in P19 cells^38^, suggesting that bilobalide can activate the Wnt pathway by cell-autonomous and autocrine/paracrine mechanisms. Here, we showed that GB-promoted neurogenesis was attenuated by knockdown of *β-catenin*, demonstrating that GB activates the Wnt pathway in postnatal NSCs in a cell-autonomous manner (Fig. 5). It will also be interesting to investigate whether GB can induce Wnt secretion in the SVZ of postnatal mice.

Free radicals have been shown to induce cell death. GBE is reported to function as a free-radical scavenger to attenuate oxidative stress^20^. Since GBE or GB did not affect cell survival in P19 cells or postnatal NSCs (Fig. 3) and we did not expose our cells with exogenous oxidative stress, it is less likely that the neurogenic effect of GBE and GB is merely mediated by acting as free-radical scavengers. Increased oxidative stress, on the other hand, has been reported to be accompanied with neuronal differentiation *in vivo* and *in vitro*^39^. Free-radical scavengers decreased neuronal differentiation in P19 cells^40^ and PC12 cells^41^. These studies further argue against the possibility that the role of GBE and GB in neuronal differentiation is through free-radical scavenging. Interestingly, high oxidative stress is associated with cell proliferation in NSCs from the SGZ^42^. Since we found that GB promoted cell cycle exit in postnatal NSCs (Fig. 3), this effect could be dependent on free-radical scavenging.

Supplement of GB has been demonstrated beneficial to the central nervous system in multiple aspects. GB protects neurons from apoptosis in AD and spinal cord injury models^30,43^. The anti-apototic effect of GB is mediated by BDNF or JAK/STAT pathway^30,43^. In addition, bilobalide prevents beta amyloid protein 1–42-induced apoptosis through the PI3K/Akt pathway^44^. Here, we provide evidence that GB promotes neurogenesis through the Wnt pathway in postnatal NSCs. Since GB can cross the blood-brain barrier^45^, supplement of GB could be a potential way to improve the condition of neurodegenerative disorders or brain injuries.

## Methods

### Chinese herbal medicines

GBE was provided from a GMP pharmaceutical company (Sun Ten Pharmaceutical Co., Ltd., Taipei, Taiwan) and diluted in water. GA or GB (Sigma) was dissolved in Dimethyl sulfoxide (DMSO).

### P19 cell culture

The mouse embryonic carcinoma cell line P19 cells were cultured in MEMα (Invitrogen) with 7.5% calf serum (HyClone), 2.5% fetal bovine serum (FBS; HyClone) and 1% Penicillin-Streptomycin-Glutamine (Invitrogen) at a 37 °C, 5% CO_2_ incubator and maintained subconfluent prior to differentiation^26^.

For neuronal differentiation, P19 cells were culture in 24-well plates coated with 2 µg/ml of mouse laminin (Invitrogen). 1 × 10^4^ of P19 cells per well were cultured in Opti-MEM (Invitrogen) containing 1% FBS and 1% antibiotics as differentiation media with GBE of different concentrations (1 ng/ml, 1 µg/ml or 1 mg/ml) for three days.

In retinoic acid (RA; Sigma) induced-neuronal differentiation, P19 cells were cultured as previously described with modifications^46^. In brief, P19 cells were cultured to form aggregates with media containing 500 nM of RA for four days first. 8 × 10^4^ of dissociated P19 cells per well were cultured as monolayer in differentiation media with GBE (1 mg/ml) for another three days. Half of the media were replaced every two days.

### Primary NSC cultures

Neurosphere cultures were prepared as previously described with modifications^47,48^. Handling of mice was according to university guidelines and the animal use protocol was approved by the Institutional Animal Care and Use Committee (IACUC) of National Taiwan Normal University (Approval Number 104037). Forebrains from postnatal day 7 (P7) CD1 mice were dissected and transferred into ice-cold Opti-MEM. SVZ tissues dissected from the lateral ventricle were minced and dissociated with trypsin. SVZ cells were cultured in 24-well plates (2 litter of mice per experiment) in SFM (DMEM/F12 and 1% N2; Invitrogen) containing 10 ng/ml bFGF (Sigma), 20 ng/ml EGF (Sigma), 2 mg/ml Heparin (Sigma) and 1% antibiotics at a 37 °C, 5% CO_2_ incubator for five days. Half of the media were replaced every two days.

For differentiation, 1 × 10^5^ cells mechanically dissociated from neurospheres were cultured in SFM with 1% FBS, 1% antibiotics without growth factors and plated in 24-well plates coated with 2 µg/ml of laminin for three days. GBE, GA, or GB was added one hour after plating. Media were replaced every two days.

For transfection, 1 × 10^5^ cells were plated in 24-well plates and cultured in SFM with 1% FBS without antibiotics for one to two hours before transfection^48^. Cells were co-transfected with 0.25 µg of *US2-GFP* and 0.35 µg of *shcat#1*, *#2* or *shLacZ* as control. Lipofectamine^TM^ 2000 (Invitrogen) was used for transfection according to the manufacturer’s instruction. Media were replaced with SFM with 1% FBS, 1% antibiotics and GB six hours after transfection. Transfected cells were cultured for three days on cover slips coated with poly-L-lysine (Sigma) and laminin (Invitrogen).

### Plasmid for knockdown experiments

*shcat#1*, *shcat#2* and *shLacZ* were obtained from the National RNAi Core Facility and the target sequences are the following: shcat#1: 5′-CCCAAGCCTTAGTAAACATAA-3′; shcat#2: 5′-GCTGATATTGACGGGCAGTAT-3′; shLacZ: 5′-CGCTAAATACTGGCAGGCGT-3′.

### Immunofluorescence

Cells were rinsed once with phosphate-buffered saline (PBS, pH 7.4) and then fixed in 4% paraformaldehyde for 15 min. After washed with 1X PBT (PBS with 0.2% Triton X-100), cells were incubated in goat serum blocking buffer for one hour prior to incubation with primary antibodies of mouse anti-TUJ1 (1:1000; Covance), mouse anti-MAP2 (1:500; Millipore), rabbit anti-Caspase3 (1:200; Cell Signaling), rabbit anti-phospho-Histone3 (1:0000; Abcam) and rabbit anti-GFP (1:1000; Invitrogen) at 4 °C for 24 hours. Labeling was visualized with DyLight^TM^ 550- or 488-conjugated goat anti-mouse or anti-rabbit IgG secondary antibody (1:1000; Abcam) at room temperature for two hours. After wash, cell nuclei were stained by DAPI (Invitrogen) for 30 min and cells were preserved in PBS containing 0.02% sodium azide or mounted with anti-fade media (Pro-Long Gold, Invitrogen). For BrdU incorporation, 5 µM of BrdU was added into the media two hours before fixation. Cells were fixed and treated with 2 N HCl at 37 °C for 30 min and 0.1 M of sodium borate for 10 min at room temperature before blocking. Cells were then incubated with rat anti-BrdU (1:500; Accurate) at 4 °C for 24 hours. Labeling was visualized with DyLight^TM^ 488-conjugated goat anti-rat IgG secondary antibody (1:1000; Abcam).

### Nuclear fractionation

P19 cells were plated in 6-well plates at 80–90% confluence in MEMα with 7.5% calf serum, 2.5% FBS and 1% Penicillin-Streptomycin-Glutamine. For nuclear fractionation, cells were treated with 20 nM of GA, GB or vehicle for three hours in the differentiation condition. Nuclear proteins were isolated by Subcellular Protein Fractionation Kit for Cultured Cells (Thermo) according to the manufacturer’s instruction.

### Western blot

For Western blot analysis, 6 × 10^5^ of P19 cells per well were plated in 6-well plates in the differentiation condition. 6 × 10^5^ of NSCs from dissociated 1’ NSs were cultured in 12-well plates. GBE, GA or GB was added one hour after plating. Three or 24 hours after treatment, cells were washed once with PBS and lysed in lysis buffer (20 mM Tris-Base, pH7.9, 20 mM aCl, 20 mM β-glycerol phosphate, 1 mM EDTA, 1 mM PMSF, 25 nM Calyculm A, 0.5% Triton and protease inhibitor cocktail). The protein concentration was determined using Bio-Rad Protein Assay. Equal amount of protein extracts (20 µg) were separated by sodium dodecylsufate-poly-acryamide gel electrophoresis (SDS-PAGE) and immunoblotting was carried out according to standard methods. Primary antibodies used for Western blot were rabbit anti-pERK (1:2500; Cell Signaling Technology), mouse anti-ERK (1:2500; Genetex), rabbit anti-β-catenin (1:500; Abcam), rabbit anti-Axin2 (1:2000; Abcam), rabbit anti-Cyclin D1 antibody (1:10000; Abcam), rabbit anti-Nucleolin (1:1000; Abcam) and mouse anti-β-tubulin (1:1000; Sigma). For immunoblotting of β-catenin (85 kDa) and Nucleolin (100 kDa) sequentially, membranes immunoblotted with anti-β-catenin were stripped with the stripping buffer (Biomate) according to the manufacturer’s instruction and immunoblotted with anti-Nucleolin. To visualize protein levels, HRP-conjugated goat anti-rabbit or goat anti-mouse secondary antibodies (1:20000; Jackson ImmunoResearch) and ECL kit (Thermo) were used. Chemiluminescent was detected by Luminescent image analyzer Las4000. Signal intensities of Western blot were quantified by using Image J.

### Luciferase assay

For the activity of Wnt/β-catenin pathway, P19 cells in 12-well plates were transfected with 0.05 µg of *US2-renilla Luciferase* and 0.5 µg of *firefly Luciferase* reporter constructs with either TCF/LEF binding site (AGATCAAAGG) or mutated binding site (AGGCCAAAGG)^49^. Six hours after transfection, media were replaced with Opti-MEM supplemented with 1% FBS and GA/GB. The reporter activity was measured 24 hours after GA/GB treatment by using the Dual-Luciferase Assay System (Promega) according to the manufacturer’s instruction. Firefly Luciferase activities were normalized with the renilla Luciferase activities to control for transfection efficiency or cell survival. Normalized Luciferase activities of the reporters with wild-type binding sites were further normalized to that of the reporters with mutated binding sites.

### Image and statistical analysis

Images were taken by an inverted fluorescence microscope or high content analysis (HCA) microscope system at 20X magnification in the Image Core at NTNU. Labeled cells were selected randomly in ten fields and counted from each sample. Statistical analyses were performed by using SPSS. Unpaired two-tailed Student’s t-test was used for two-group comparisons. One-way or two-way ANOVA followed by Tukey’s *post hoc* test was used for multiple comparisons. Results are shown as mean ± standard error of the mean (SEM); significance level was p < 0.05.

## Electronic supplementary material

Supplementary information

## Data Availability

All data generated or analysed during this study are included in this published article.
