# Supplementary material for: Ginkgolide B promotes neuronal differentiation through the Wnt/β-catenin pathway in neural stem cells of the postnatal mammalian subventricular zone
Source: Sci Rep. 2018 Oct 8;8:14947. doi: 10.1038/s41598-018-32960-8 (PMC6175824; doi:10.1038/s41598-018-32960-8)
Supplement: Supplementary file 1 — Supplementary information [file 41598_2018_32960_MOESM1_ESM.docx]

**Supplemental information**

**Ginkgolide B promotes neuronal differentiation through the Wnt/β-catenin pathway in neural stem cells of the postnatal mammalian subventricular zone**

Ming-Yang Li^1^, Chia-Ting Chang^1^, Yueh-Ting Han^1^, Chien-Po Liao^1^, Jenn-Yah Yu^2, 3^, and Tsu-Wei Wang^1*^

*^1^Department of Life Science, National Taiwan Normal University, Taipei 116, Taiwan*

*^2^Department of Life Sciences and Institute of Genome Sciences, National Yang-Ming University, Taipei 112, Taiwan*

*^3^Brain Research Center, National Yang-Ming University, Taipei 112, Taiwan*

^*^Corresponding author: TW Wang ([twwang@ntnu.edu.tw](mailto:twwang@ntnu.edu.tw)*)*

**
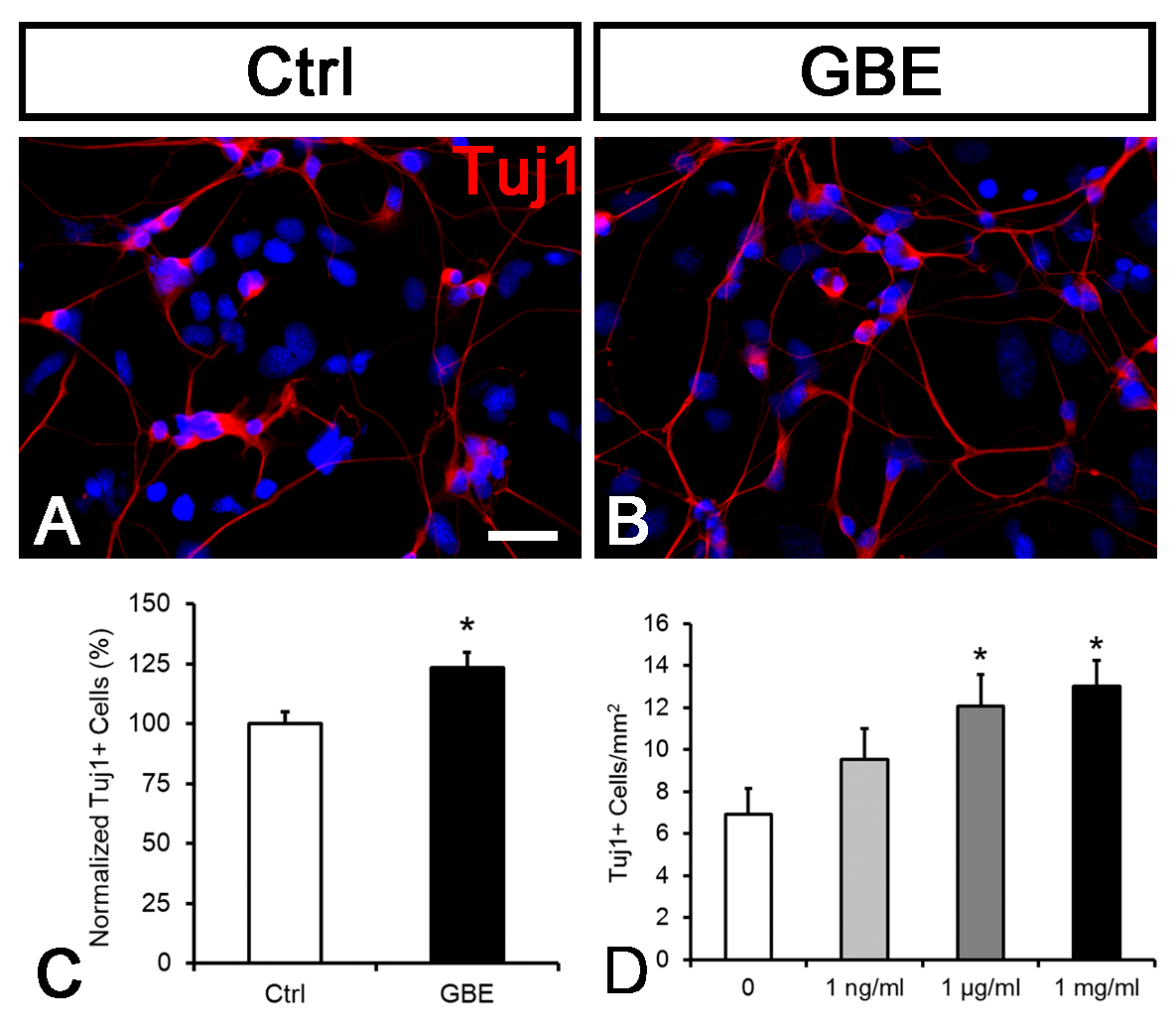
**

**Supplementary Figure 1. GBE promotes P19 cells to differentiate into neurons.** (A, B) After being cultured as aggregates with RA for four days, P19 cells were dissociated and cultured in the differentiation condition with vehicle (Ctrl) or 1 mg/ml of GBE for another three days. Differentiating and differentiated neurons were labeled with Tuj1 antibodies in red. Nuclear DNA was stained with DAPI in blue. (C) Quantification analysis of Tuj1-positive cell numbers. GBE significantly increased Tuj1-positive cells. The rate of neuronal differentiation was normalized to that of the control. n=3, t-test. (D) P19 cells were cultured in the differentiation condition with vehicle or various concentrations of GBE for three days and immunolabeled with Tuj1. 1 µg/ml and 1 mg/ml of GBE significantly increased Tuj1-positive cells. n=3, one-way ANOVA followed by Tukey’s *post hoc* test. All data are shown as mean ± SEM. *: *p* < 0.05 compared to the control group. Scale bar: 50 µm.

**Supplementary Figure 2. The original gel of Figure 4A.** Proteins of the control (Ctrl), GA and GB group from the cytosol and nucleus were loaded on the left and right part of the gel, respectively. The membrane was cut into the upper and the lower part for Western blotting of β-catenin (85 kDa)/nucleolin (100 kDa) and β-tubulin (55 kDa), respectively. The upper membrane was immunoblotted with anti-β-catenin first, stripped and immunoblotted with anti-nucleolin. Protein marker was in the middle of the membrane.

**Supplementary Figure 3. The original gel of Figure 4E.** The membrane was cut into the upper and the lower part for Western blotting of β-tubulin (55 kDa) and Cyclin D1 (34 kDa), respectively. Proteins from the control (Ctrl), GA and GB group were loaded on the right part of the gel. Protein marker was in the middle of the membrane. Red box areas were selected for the quantification of protein expression and shown in Figure 4E.


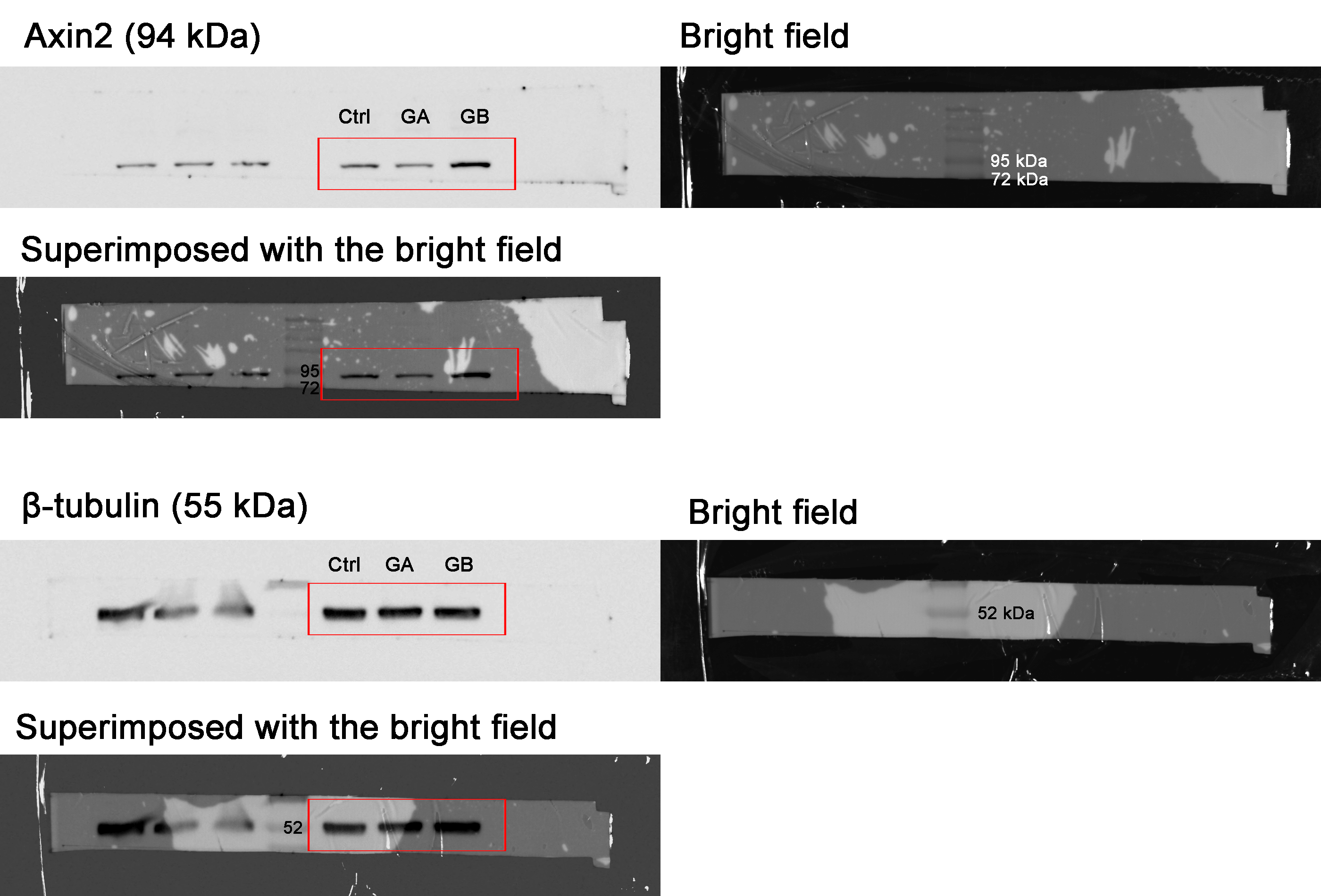


**Supplementary Figure 4. The original gel of Figure 4G.** The membrane was cut into the upper and the lower part for Western blotting of Axin2 (94 kDa) and β-tubulin (55 kDa), respectively. Proteins from the control (Ctrl), GA and GB group were loaded on the right part of the gel. Protein marker was in the middle of the membrane. Red box areas were selected for the quantification of protein expression and shown in Figure 4G.


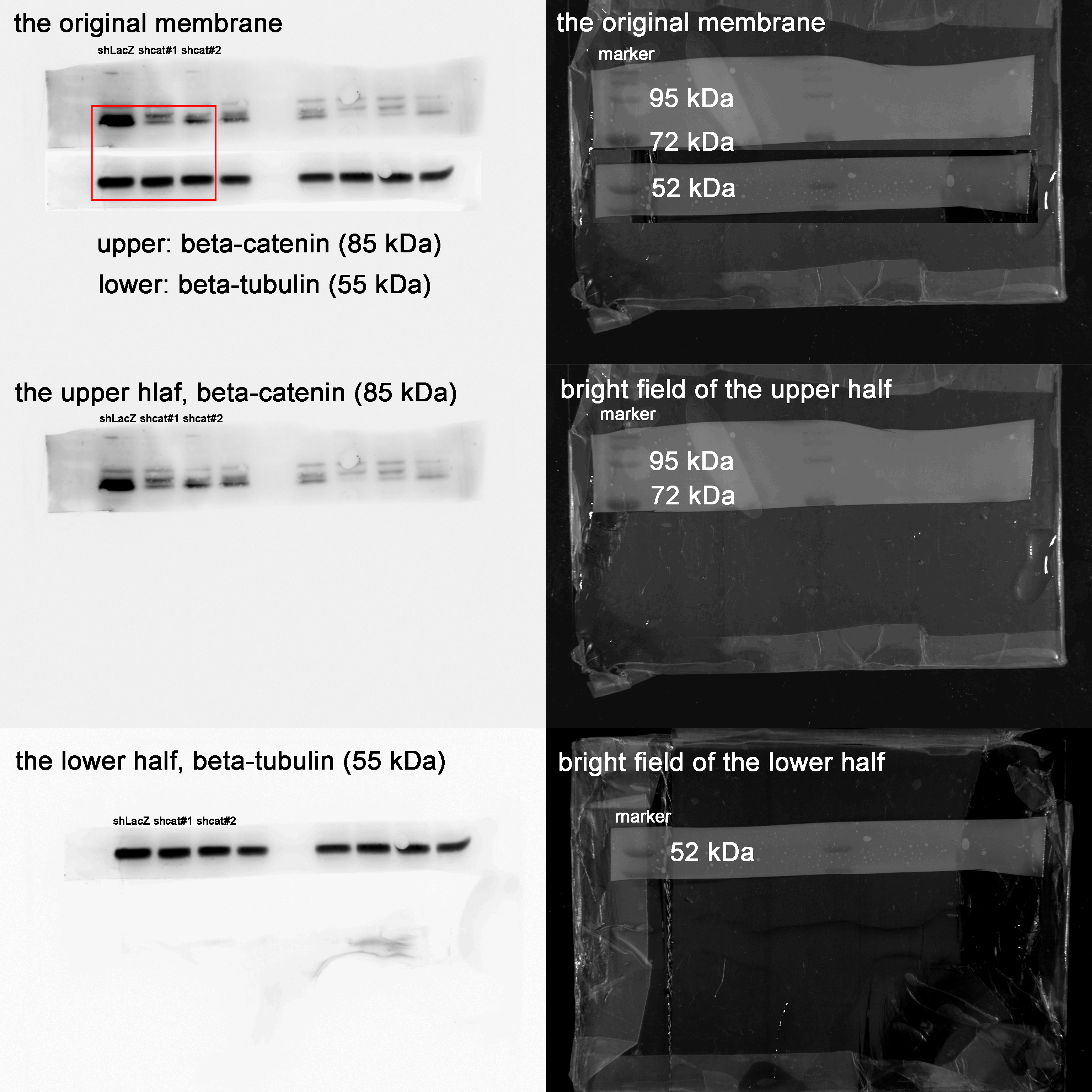


**Supplementary Figure 5. The original gel of Figure 5A.** The membrane was cut into the upper part and the lower part for Western blotting of β-catenin (85 kDa) and β-tubulin (55 kDa), respectively. Proteins were loaded on the left part of the gel. The left column was the shLacZ control group; the second and third column from the left was shcat#1 and shcat#2 group, respectively. Protein marker was on the left side and the middle of the membrane. Red box areas were selected for the quantification of protein expression and shown in Figure 5A.
